# Non-coding RNA profiles associated with equine osteoarthritis: a systematic review

**DOI:** 10.3389/fvets.2026.1917722

**Published:** 2026-08-26

**Authors:** Elham Khaliji, Talia Sonnenschein Wong, Christopher Michael Barker, Shuai Chen, Jennifer Michelle Cassano

**Affiliations:** 1 Department of Veterinary Medicine and Epidemiology, Veterinary Institute for Regenerative Cures, Weill School of Veterinary Medicine, University of California, Davis, CA, United States; 2 Department of Pathology, Microbiology and Immunology, Center for Vector-borne Diseases, Weill School of Veterinary Medicine, University of California, Davis, CA, United States; 3 Department of Public Health Sciences, School of Medicine, University of California, Davis, CA, United States

**Keywords:** biomarker, differential expression, equine, microRNA, next generation sequencing (NGS), non-coding RNA, osteoarthritis

## Abstract

**Introduction:**

Non-coding RNAs (ncRNAs) play a role in the pathogenesis of osteoarthritis (OA) by regulating gene expression related to inflammation, chondrocyte apoptosis, and extracellular matrix degradation. In horses, OA is a leading cause of lameness and poor performance, with no current treatments halting disease progression and no clear biomarker available. Although some studies have investigated expression profiles of ncRNAs in equine OA, there is a need to systematically review previous efforts and synthesize information to identify potential ncRNAs to target in future research.

**Methods:**

The search strategy was developed using the Population, Intervention, Comparison, Outcome, Studies (PICOS) framework. A comprehensive search of Scopus, ScienceDirect, CAB Abstracts, BIOSIS Previews, and Web of Science Core Collection identified studies written in English published through June 30, 2025, using comprehensive terms for ncRNAs in equine osteoarthritis. Eligible studies assessed differential ncRNAs expression profiles in natural and experimentally induced OA and control equids. Individual ncRNAs were categorized based on frequency across different studies: 1) identified in three or more samples across studies, 2) identified in two samples across studies or 3) unique to one sample in a single study. Differential expression (log_2_fold change) with *p*-values were extracted for each ncRNA and visualized using heatmaps.

**Results:**

Eight studies met the inclusion criteria, including four longitudinal and four case-control studies. Three investigated natural OA, four analyzed surgically induced OA and one employed a chemically induced OA model. Most studies utilized next-generation sequencing (NGS) to analyze ncRNAs, while two used quantitative polymerase chain reaction (qPCR). Three NGS studies included PCR validation. Multiple ncRNAs were identified, with microRNAs (miRNAs) being the most frequently found, followed by small nucleolar RNAs (snoRNAs). Among ncRNAs reported in at least three types of samples across independent studies, let-7a, miR-1307, miR-144, miR-744, and miR-98 were significantly upregulated, whereas miR-10a and miR-29b were significantly downregulated (*p* < 0.05).

**Discussion:**

Our findings of specific ncRNAs with shared expression profiles across multiple equine OA studies support their potential as OA biomarkers. Further research is necessary to determine the role of ncRNAs in OA development, such as contributions to cartilage degeneration, inflammation, and apoptosis, and to evaluate their potential as therapeutic targets.

## 1 Introduction

Osteoarthritis (OA) is a complex heterogenous disease with multifactorial etiology, including trauma or overuse, abnormal joint conformation, and age (1). OA is a disease of the entire joint and involves synovial inflammation, thickening of the joint capsule, ligament degeneration, subchondral bone sclerosis, and irreversible destruction of the articular hyaline cartilage (2–4). Joint disease, including OA, is one of the primary causes of lameness in horses (5). Survey estimates that around 60% of equine lameness is related to OA (6). It is the most common reason for lost days of training and early retirement, which may lead to euthanasia (1, 7–9).

The pathophysiology of OA remains incompletely understood (10). Equine OA is usually diagnosed by imaging, which detects changes only after the disease has advanced and osteophytes or substantial cartilage damage have developed (11). Advanced OA is characterized by more severe structural damage, including increased bone loss (12), marginal osteophyte formation, subchondral bone irregularity (13) compared to early OA. By this stage, the changes are irreversible despite currently available treatments due to the limited healing capacity of articular cartilage (14). Advancing methods for early detection, developing more effective treatments, and deepening our understanding of OA pathogenesis are needed (15).

Non-coding RNAs (ncRNAs) comprise approximately 99% of total cellular RNA content and, together with DNA methylation and histone modifications, form a key epigenetic regulatory mechanism (16). They are not translated into proteins but instead regulate the expression of specific gene targets (17).

Dysregulation of ncRNA associated with cancer, cardiovascular, neurological disorders, infectious disease, and sepsis in human (18). In addition, they are essential for regulating biological processes involved in joint development (19), as well as response to injury and repair (20). Dysregulation of ncRNAs contributes to the pathogenesis of joint disease, including OA (21, 22), highlighting candidate targets for diagnosis and treatment.

NcRNAs shorter than 200 nucleotides are classified as short ncRNAs, a group in which miRNAs have been the most widely investigated. MiRNAs are produced through a multi-step process in which primary transcripts (pri-miRNA) are processed within the nucleus to form precursor miRNAs (pre-miRNAs) and then transported to the cytoplasm for maturation. The resulting miRNAs are typically 18–24 nucleotides long and modulate gene expression by inducing mRNA degradation or translational repression (23).

SnoRNAs are a class of ncRNA 60–300 nucleotides in length (23), predominantly localized to the nucleolus and primarily encoded within introns of protein-coding and non-coding genes (24). Maintenance of cartilage homeostasis requires sustained synthesis of ECM components, which depends on precise regulation of ribosome biogenesis, composition, and function (25). As snoRNAs are essential regulators of rRNA processing and modification, dysregulation of snoRNA expression may alter ribosomal function and chondrocyte proteomes (26, 27).

Despite ongoing research and growth in publications on ncRNAs associated with equine OA, a holistic synthesis of published evidence is lacking. In this study we sought to determine whether ncRNAs associated with OA exhibit shared expression profiles across published studies. The first aim was to provide a comprehensive analysis of the existing literature on ncRNA expression in equine OA. The second aim was to synthesize differential expression of ncRNAs between OA and normal joints using log_2_ (Fold Change) from published studies. The findings are expected to inform future research supporting the development of more precise diagnostic platforms and treatment strategies for OA in horses.

## 2 Methods

### 2.1 Inclusion and exclusion criteria

The inclusion and exclusion criteria for this review were based on the PICOS framework. The inclusion criteria were as follows,

**P**opulation: Horses with natural and experimentally induced OA.

**I**ntervention: both observational studies with no treatment and experimental studies with treatment.

**C**omparison: equine *in vivo* controls without OA or sham-injected joints.

**O**utcome(s): differential expression of ncRNAs (e.g., fold-change or log_2_ (fold change), with statistical significance).

**S**tudies: peer-reviewed publications, published through Jun 2025.

The following were excluded from the review: studies published in languages other than English, duplicate data, non-equine studies, *in-vitro* studies, non-OA studies, review articles, and conference abstracts.

### 2.2 Search strategy

Based on the determined inclusion and exclusion criteria, two researchers independently screened the retrieved literature records. An example of the search strategy used in PubMed is provided below. Comparable search strategies were employed for other databases, including Scopus, ScienceDirect, CAB Abstracts, BIOSIS Previews, and the Web of Science Core Collection.

(“RNA, Untranslated”[MeSH] OR “MicroRNAs”[MeSH] OR “RNA, Small Nucleolar”[MeSH] OR “RNA, Small Nuclear”[MeSH] OR“RNA, Transfer”[MeSH] OR “RNA, Ribosomal”[MeSH] OR “RNA, Long Noncoding”[MeSH] OR “piRNA”[tiab] OR “piRNAs”[tiab] OR “PIWI-interacting RNA”[tiab] OR “non-coding RNA”[tiab] OR “ncRNA”[tiab] OR “miRNA”[tiab] OR “microRNA”[tiab] OR“long non-coding RNA”[tiab] OR “lncRNA”[tiab] OR“circular RNA”[tiab] OR “snoRNA”[tiab] OR “snoRNAs”[tiab] OR “snRNA”[tiab] OR “snRNAs”[tiab] OR “sncRNA”[tiab] OR “small non-coding RNA”[tiab] OR “small nuclear RNA”[tiab] OR “tRNA”[tiab] OR “transfer RNA”[tiab] OR “rRNA”[tiab] OR “ribosomal RNA”[tiab] OR “scaRNA”[tiab] OR “scaRNAs”[tiab] OR “exRNA”[tiab] OR “exRNAs”[tiab] OR “Y RNA”[tiab] OR “Y-RNA”[tiab] OR “Y RNAs”[tiab] OR “Xist”[tiab] OR “HOTAIR”[tiab])

AND

(“Equidae”[MeSH] OR “Horses”[MeSH] OR horse[tiab] OR horses[tiab] OR equine[tiab] OR equines[tiab])

AND

(“Osteoarthritis”[MeSH Terms] OR “Synovitis”[MeSH Terms] OR “Arthritis”[MeSH Terms] OR osteoarthritis[tiab] OR OA [tiab] OR arthritis[tiab] OR synovitis[tiab] OR “joint disease”[tiab] OR (joint^*^[tiab] AND disease^*^[tiab]) OR “cartilage degeneration“[tiab] OR ”Inflammatory arthritis“[tiab])

**Figure 1:**
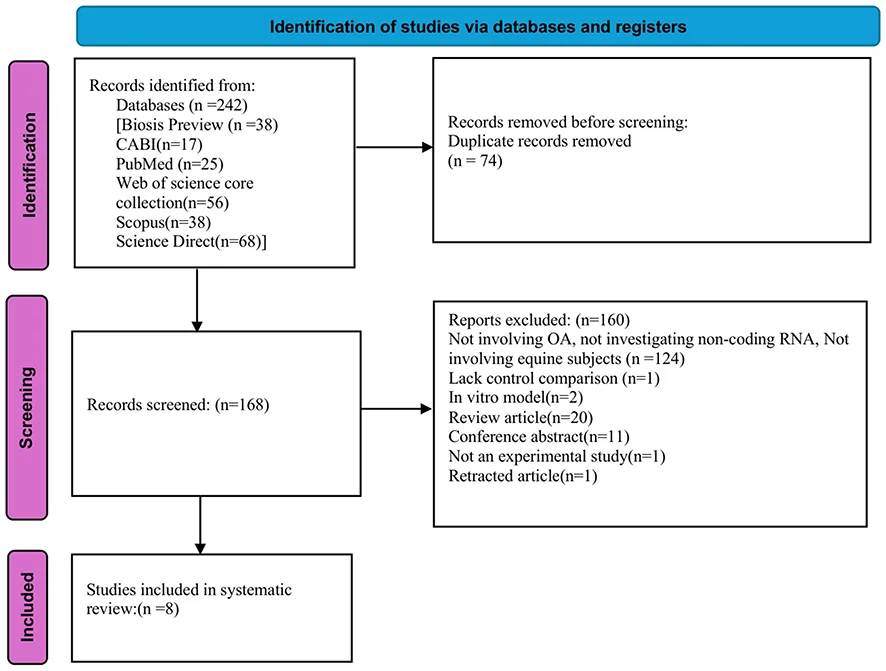
PRISMA flow diagram. The flow diagram shows the number of records identified, included and excluded, and the reasons for exclusions. Adapted From: Page et al. (28), Licensed under CC BY 4.0.

All retrieved search results were imported into Sciwheel, where duplicate records were removed. The inclusion and exclusion criteria were then applied independently by two reviewers (EK and TSW). Any disagreements were resolved through discussion, with input from a third reviewer (JMC) when necessary. A PRISMA flow diagram illustrates the screening process (Figure 1) (28).

### 2.3 Data extraction

For studies that met all inclusion criteria, relevant data were extracted and summarized as follows: study characteristics included study, year, country, study design, sample type, species, OA model, OA diagnosis, subjects (number of cases and number of controls), subject weight, age, breed and sex (Table 1), non-coding RNA analysis workflow which included ncRNA isolation and library preparation, sequencing, Polymerase Chain Reaction (PCR) validation and analysis (Table 2).

**Table 1:** Study characteristics.

References	Country	Study design	Sample type	Species	OA model	OA diagnosis	Subjects (number of cases)	Subjects (number of controls)	Weight	Age (years)	Breed	Sex
Andersen et al. (15)	Denmark (University of Copenhagen)	Case-Control	Synovial Membrane & Cartilage	Equine	Induced OA (Osteochondral fragment model)	Gross	*n* = 9 (untreated OA, left middle carpal)	*n* = 8 (untreated control, right middle carpal)	396–535kg (mean: 472 kg)	3–7	Standard-bred trotters	Mare (15), Gelding (2)
Anderson et al. (32)	Denmark (University of Copenhagen)	Longitudinal	Plasma. EV & Synovial Fluid. EV	Equine	Induced OA (Osteochondral fragment model)	Gross & Histopathologic	*n* = 4 (left middle carpal)	n=4 (right middle carpal)	397–470kg	2.5-7	Standard-bred trotters	Not Stated
Antunes et al. (35)	Canada (Ontario Veterinary College)	Case-Control	Plasma & Synovial Fluid	Equine	Natural OA	Radiographic & Arthroscopic	*n* = 5 OA (*n* = 3 radiocarpal, *n* = 1 metacarpophalangeal, *n* = 1 metatarsophalangeal)	*n* = 4 controls (*n* = 2 tibiotarsal, *n* = 1 metatarsophalangeal, *n* = 1 metacarpophalangeal)	Not Stated	Control: 17 ± 3.3 OA: 3.29 ± 0.7	Control: Clydesdale Cross (1), Draft Cross (1), Thoroughbred (1), Standard-bred (1) OA: Thoroughbred (1), Standardbred (2), Quarter (1)	Control: Female (3), Male (1), Male Castrated (0). OA: Female (1), Male (1), Male Castrated (3)
Castanheira et al. (36)	United Kingdom (University of Liverpool)	Case-Control	Synovial Fluid	Equine	Natural OA	Gross & Histopathologic	*n* = 6 (metacarpophalangeal)	*n* = 6 metacarpophalangeal	Not Stated	Control: 22 ± 2 OA: 22 ± 7.5	Not Stated	Not Stated
Chabronova et al. (34)	Denmark (University of Copenhagen)	Longitudinal	Synovial Fluid	Equine	Induced OA (Osteochondral fragment model)	Histopathologic & Radiographic	*n* = 9 (left middle carpal)	*n* = 9 (right middle carpal)	397–528kg	2.5–7	Standard-bred trotters	Mare (7), Gelding (2)
Connard et al. (37)	United States (University of Pennsylvania)	Case-Control	Plasma. EV& Synovial Fluid. EV	Equine	Natural Post-Traumatic OA	Gross	*n* = 6 Post-traumatic OA (*n* = 1 radiocarpal, *n* = 3 middle carpal, *n* = 2 metacarpophalangeal)	*n* = 6 control (*n* = 2 radiocarpal, *n* = 2 middle carpal, *n* = 2 metacarpophalangeal)	Not Stated	Control: 4 ± 1.53 OA: 7 ± 1.91	Thoroughbred	Control: Filly: (2), Mare: (1), Gelding: (3) PTOA: Mare: (2), Gelding: (4)
Walters et al. (33)	Denmark (University of Copenhagen)	Longitudinal	Synovial Fluid	Equine	Induced OA, (Osteochondral fragment model)	Gross & Histopathologic	*n* = 9 (left middle carpal)	*n* = 9 (right middle carpal)	397–528kg	2.5–7	Standard-bred trotters	Mare (7) Gelding (2)
Yassin et al. (30)	Egypt (University of Cairo)	Longitudinal	Synovial Fluid & Serum	Donkey	Chemically induced OA [25mg/ml Sodium Monoidoaceate (MIA)]	Gross & Histopathologic Lameness evaluation Radiographic Ultrasonographic	*n* = 9 (left radiocarpal)	*n* = 9 (left radiocarpal time0 before OA induction)	150–200 kg	3–5	Local	Male

The characteristics across studies were extracted including references, country, study design, sample type, species, OA model, OA diagnosis, subject (number of cases and controls), weight, age, breed, sex.

**Table 2:** Non-coding RNA analysis workflow.

References	Non-coding RNA isolation and library preparation					Sequencing			PCR validation			Analysis	
	Hyaluronidase used	Sample processing (RNA extraction)	RNA integrity assessment	Total RNA extracted	RNA concentration for library prep	Sequencing performed (yes/no)	Sequencing platform	Sequencing type	PCR validation (yes/no)	miRNAs validated	Sample type	DE	Statistical adjustment for multiple testing
Andersen et al. (15)	Not stated	miRNeasy mini kit (Qiagen)	Agilent 2100 Bioanalyzer (Agilent Technologies, Nærum, Denmark) using the RNA 6000Nano Kit.	Not Stated	N/A	No	N/A	N/A	Expression measured directly by RT-qPCR	N/A	Cartilage & Synovial membrane	Log_2_FC	None reported
Anderson et al. (32)	1 μg/ml	miRNeasy serum/plasma advanced kit (Qiagen, Crawley, United Kingdom)	Not stated	Not Stated	2μl	Yes	NovaSeq SP100 flow cell (Illumina Sandiego, United States)	Small RNA-seq	No	N/A	N/A	Log_2_FC	FDR (method not specified)
Antunes et al. (35)	Not stated	serum/plasma advanced kit (Qiagen, Hilden, Germany)	Not stated	Extracted plasma miRNA (5μl)	Extracted plasma miRNA (5μl)	Yes	NextSeq 550 using NextSeq 500/550 High Output Kit v2.5 at the Schroeder Arthritis Institute (Toronto)	miRNA	Yes (ddPCR)	miR-140-5p miR-181a miR-196b miR-20a miR-486-3p	Plasma & Synovial Fluid	Log_2_FC	FDR (Benjamini–Hochberg)
Castanheira et al. (36)	1 μg/ml	miRNeasy serum kit (Qiagen, Crawley, UK)	Agilent 2100, Bioanalyzer system using on RNA Pico Chip	Not Stated	100 ng	Yes	Illumina High seq 4,000 (New England Biosciences (NEB), Ipswich, USA)	Small RNA-seq	Yes (RT-qPCR)	miR-143 miR-223 miR-99a miR-23b let-7a-2 snord96A snord13	Synovial Fluid	Log_2_FC	FDR (method not specified)
Chabronova et al. (34)	500μl Synovial fluid+ Hyaluronidase (1 μg/ml)	miRNeasy serum/plasma advanced kit (Qiagen, Manchester, UK)	RNA qualities were determined by a Nanodrop and validated using Qubit Flex Fluorometer, and RIN scoring	Not Stated	100 ng/sample	Yes	Illumina Hiseq 4,000 using 2 x 150 bp ends	Small RNA-seq	No	N/A	N/A	Log_2_FC	FDR (Benjamini–Hochberg)
Connard et al. (37)	10 μg/ml	Quik-RNA miniprep kit (Zymo Research Corporation).	Agilent 2100 Bioanalyzer and RNA 6000 Pico Kit (Agilent Technologies)	Not Stated	Not Stated	Yes	Illumina	Small RNA-seq	No	N/A	N/A	Log_2_FC	FDR (method not specified)
Walters et al. (33)	500μl Synovial fluid+ Hyaluronidase (1 μg/ml)	miRNeasy serum/plasma advanced kit (Qiagen)	Not Stated	Not Stated	100 ng/sample	Yes	Illumina High Seq 4,000	Small RNA-seq	Yes (RT-qPCR)	miRNA- 199b-3p miRNA-139-5p mirNA-1839 miRNA-151-5p	Synovial Fluid	Log_2_FC	None Reported
Yassin et al. (30)	Not stated	miRNeasy mini kit (Qiagen, Hilden, Germany)	Nanodrop ND-1000	20μl	N/A	No	N/A	N/A	Expression measured directly by RT-qPCR	N/A	Synovial Fluid Serum	2^−Δ*ΔCT*^	None reported

Methodology of ncRNA isolation, ncRNA library preparation, sequencing, PCR validation, and analysis from eligible studies were extracted. N/A, Not applicable. △△ is a method used to determine the relative foldchange in gene expression between treatment samples and control samples.

Additionally, data on log_2_ (fold change) comparing diseased and normal samples were extracted from the eligible studies, along with the *p*-values for subsequent analysis. The data were retrieved from published papers, supplementary materials or by contacting the corresponding authors.

### 2.4 Methodological quality of all eight included studies

The methodological quality of all eight included studies was assessed using the Newcastle-Ottawa Scale (NOS). The NOS evaluates three domains: selection, comparability, and exposure. Potential attrition bias was also considered during the quality assessment. A maximum of nine points were assigned based on methodological features that reduced the potential for bias. Studies with NOS scores of 7–9 were considered to have a low risk of bias.

### 2.5 Evidence synthesis

The ncRNAs were divided into three categories: 1) non-coding RNAs expressed in three or more samples across studies. 2) non-coding RNAs expressed in two samples across studies. 3) non-coding RNAs which were unique to one sample in a single study. Reported *p*-values and fold- change values were extracted directly from the original studies and visualized descriptively. In Andersen et al. (15) study we calculated the log_2_ (fold change) and *p*-values by comparing the ncRNA expression values between the untreated-OA and control groups. No additional statistical testing or multiple-comparisons adjustments were performed, as analytical pipelines and adjustment methods varied across studies. Reported *p*-values reflect study-specific analyses and are not intended to be directly comparable across studies.

All the statistical analysis were conducted in R version 4.3.3 (29). Heat maps were generated to visualize the expression levels of ncRNAs in log_2_ (fold change) across the studies.

## 3 Results

### 3.1 Screening results

An initial search yielded 242 publications. Following the removal of duplicates, 168 titles and abstracts remained. Full-text articles were then reviewed for eligibility, resulting in the exclusion of 160 studies (28) (Figure 1). In total, 8 studies were included in the final systematic review.

### 3.2 Study characteristics

All eight included studies were published from 2021 onward (Table 1). Four of these studies used longitudinal designs, in which samples were taken repeatedly from the same individuals over time to examine how ncRNAs expression levels varied with OA progression. The remaining four studies employed case-control designs, where expression levels of ncRNAs were measured at a single time point and compared between OA and normal joints. Although sampled tissue types varied across studies, synovial fluid was the most analyzed sample type (five studies). Seven studies were conducted in horses, and one study in donkeys (30). Three studies investigated natural OA, while four utilized a surgically induced OA model, specifically the equine carpal osteochondral fragment model (31). The study in donkeys employed a chemically induced OA model using sodium monoiodoacetate (MIA; 25mg/ml). In all studies, OA diagnosis was based on macroscopic and microscopic evaluations. Subject characteristics, including weight, age, breed, and sex, varied across studies (Table 1).

### 3.3 Data extraction

For each longitudinal study, we considered the final time point in OA-induced joints as the OA case, and the baseline sample, prior to OA induction, was used as the control. In the longitudinal analysis, OA induction occurred after time 0, and no treatment was administered at subsequent time points. Therefore, we assumed that animals exhibiting OA characteristics at the final time point represented confirmed OA cases. In addition, each longitudinal studies used different follow up time points; so, it was not feasible to select a uniform intermediate time point across studies. Based on this rationale, and to increase confidence in case classification, we used the final time point as OA cases. We extracted the data to compare the baseline sample (day 0 before OA induction) to day 63 (32) or day 70 (33, 34) after surgical induction and 7 months after MIA induction (30) (Table 3). Therefore, ncRNAs expressed at mid-study time points during OA progression were not evaluated in our analysis. This approach facilitated the integration of longitudinal studies with case-control studies. In all longitudinal studies, samples collected at day 0 prior to OA induction were considered normal joints as defined by clinical examination.

**Table 3:** Longitudinal studies' timelines.

References	OA model	Duration	Time points	OA induction	Sample collection
Anderson et al. (32)	Induced OA (Osteochondral fragment model)	63 days	Day 0, 10, 35, 42, 49, 56, 63	After day 0	Day 0, 10, 35, 42, 49, 56, 63 from OA and control
Chabronova et al. (34)	Induced OA (Osteochondral fragment model)	70 days	Day 0, 28, 70	After day 0	Day 0, 28, 70 from OA and control
Walters et al. (33)	Induced OA (Osteochondral fragment model)	70 days	Day 0, 14, 17, 21, 28, 35, 42, 49, 56, 63, 70	After day 0	Day 0, 14, 17, 21, 28, 35, 42, 49, 56, 63, 70 from OA and control
Yassin et al. (30)	Chemically induced OA [25mg/ml Sodium Monoidoaceate (MIA)]	7 months	Day 0, 1 week, 1 month, 2 months, 3 months, 5 months, 7 months	After day 0	Day 0, 1 week, 1 month, 2 months, 3 months, 5 months, 7 months from left radiocarpal

The samples were collected at day 0 before the OA induction and subsequent time points following OA induction.

The remaining four studies utilized a case-control design. Synovial fluid samples were collected from defined control joints and OA joints 70 days after OA induction (15), natural OA joints (35), early OA MCP joints (36), and post-traumatic OA joints (37). Peripheral blood samples were also collected from the patients with natural OA joints (35) and with post-traumatic OA joints (37).

### 3.4 Non-coding RNA analysis workflow

Six studies performed next generation sequencing (NGS) of ncRNAs and two studies (15, 30) utilized qPCR. Among the studies that used NGS, three studies conducted qPCR (33, 36) or droplet digital PCR (ddPCR) (35). All studies reported relative expression as fold change (Tables 2, 4).

**Table 4:** Comparison of select ncRNA expression profiles between NGS and PCR validation.

Study	miRNA	Tissue type	NGS	PCR validation
Antunes et al. (35) ddPCR validation	miR-20a	Plasma	↑OA vs. control	↓OA vs. control
	miR-181a	Plasma	↑OA vs. control	↓OA vs. control
	miR-196b	Plasma	↑OA vs. control	↓OA vs. control (no significance)
	miR-486	Plasma	↑OA vs. control	↓OA vs. control
	miR-140	Plasma	↑OA vs. control	↓OA vs. control
Castanheira et al. (36) qPCR validation	miR-143	Synovial fluid	↓OA vs. control	↑OA vs. control (no significance)
	miR-223	Synovial fluid	↓OA vs. control	↓OA vs. control
	miR-99a	Synovial fluid	↓OA vs. control	↑OA vs. control (no significance)
	miR-23b	Synovial fluid	↑OA vs. control	↑OA vs. control
	let7a-2	Synovial fluid	↑OA vs. control	↑OA vs. control
	snord96A	Synovial fluid	↑OA vs. control	↑OA vs. control
	snord13	Synovial fluid	↑OA vs. control	↑OA vs. control
Walters et al. (33) qPCR validation	miR-199b-3p	Synovial fluid	↑OA vs. control	↑OA vs. control
	miR-139-5p	Synovial fluid	↑OA vs. control	↑OA vs. control
	miR-1839	Synovial fluid	↑OA vs. control	↑OA vs. control
	miR-151-5p	Synovial fluid	↑OA vs. control	↑OA vs. control

### 3.5 Methodological quality of all eight included studies

All eligible studies had quality assessment scores ranging from 7 to 9 and were therefore considered to have a low risk of bias (Table 5).

**Table 5:** Methodological quality assessment for eight included studies.

References	Selection				Comparability	Exposure			Score
	Is the case definition adequate	Representativeness of the cases	Selection of controls	Definition of controls	Comparability of cases and controls on the basis of the design or analysis	Ascertainment of exposure (assessment of ncRNA expression)	Same method of ascertainment for cases and controls	Attrition bias	
Andersen et al. (15)	Yes (Cases defined by imaging, macroscopic and histopathologic findings) ^*^	Obviously representative series of cases (Cases were samples with experimentally untreated carpal OA) ^*^.	Controls come from same population as the cases ^*^	Controls were the horses without the disease^*^	The groups were similar in age, and weight ^**^	Measured by validated laboratory methods. ^*^	Same method of ascertainment ^*^	One horse from untreated group excluded.	8
Anderson et al. (32)	Yes (Cases defined by clinical examination, imaging, hematological and blood-biochemical analysis, and arthrocentesis) ^*^	Obviously representative series of cases (Cases were samples with surgically induced OA) ^*^.	Controls come from same population as the cases ^*^	Controls were the horses without the disease ^*^	The groups were similar in age, and weight. ^**^	Measured by validated laboratory methods. ^*^	Same method of ascertainment ^*^	No evidence of attrition bias^*^	9
Antunes et al. (35)	Yes (Cases defined by radiographic and arthroscopic assessment) ^*^	Obviously representative series of cases (Cases were samples from horses undergoing joint arthroscopy) ^*^	Controls come from same population as the cases ^*^	Controls were the horses without the disease^*^	The groups were different in breed and joints.	Measured by validated laboratory methods. ^*^	Same method of ascertainment ^*^	No evidence of attrition bias^*^	7
Castanheira et al. (36)	Yes (Cases defined by macroscopic and microscopic score) ^*^	Obviously representative series of cases (Cases have macroscopic and microscopic OA sign) ^*^	Controls come from same population as the cases ^*^	Controls were the horses with minor age-related macroscopic or histological changes ^*^	The groups were age-matched to account for any age-related changes. ^*^	Measured by validated laboratory methods. ^*^	Same method of ascertainment^*^	No evidence of attrition bias^*^	8
Chabronova et al. (34)	Yes (Cases defined by clinical examination, imaging, hematological and blood-biochemical analysis, and arthrocentesis) ^*^	Obviously representative series of cases (Cases were samples with surgically induced OA) ^*^.	Controls come from same population as the cases ^*^	Controls were the horses without the disease ^*^	The groups were similar in age and weight. ^**^	Measured by validated laboratory methods. ^*^	Same method of ascertainment ^*^	No evidence of attrition bias^*^	9
Connard et al. (37)	Yes (Cases defined by macroscopic score) ^*^	Obviously representative series of cases (Cases were samples with Gross evaluation of OA) ^*^.	Controls come from same population as the cases ^*^	Controls were the horses without the disease ^*^	The groups were different in age.	Measured by validated laboratory methods. ^*^	Same method of ascertainment ^*^	No evidence of attrition bias^*^	7
Walters et al. (33)	Yes (Cases defined by clinical examination, imaging, hematological and blood-biochemical analysis, and arthrocentesis) ^*^	Obviously representative series of cases (Cases were samples with surgically induced OA) ^*^.	Controls come from same population as the cases ^*^	Controls were the horses without the disease^*^	The groups were similar in breed, age, and weight. ^**^	Measured by validated laboratory methods. ^*^	Same method of ascertainment ^*^	Some samples lost during the study time frame	8
Yassin et al. (30)	Yes (Cases defined by clinical examination) ^*^	Obviously representative series of cases (Cases were samples with chemically induced OA) ^*^.	Controls come from same population as the cases ^*^	Controls were the horses without the disease^*^	The groups were similar in breed, age, weight, and sex. ^**^	Measured by validated laboratory methods. ^*^	Same method of ascertainment ^*^	No evidence of attrition bias^*^	9

^*^indicates points earned in that category

### 3.6 Distribution of non-coding RNA classes

**Figure 2:**
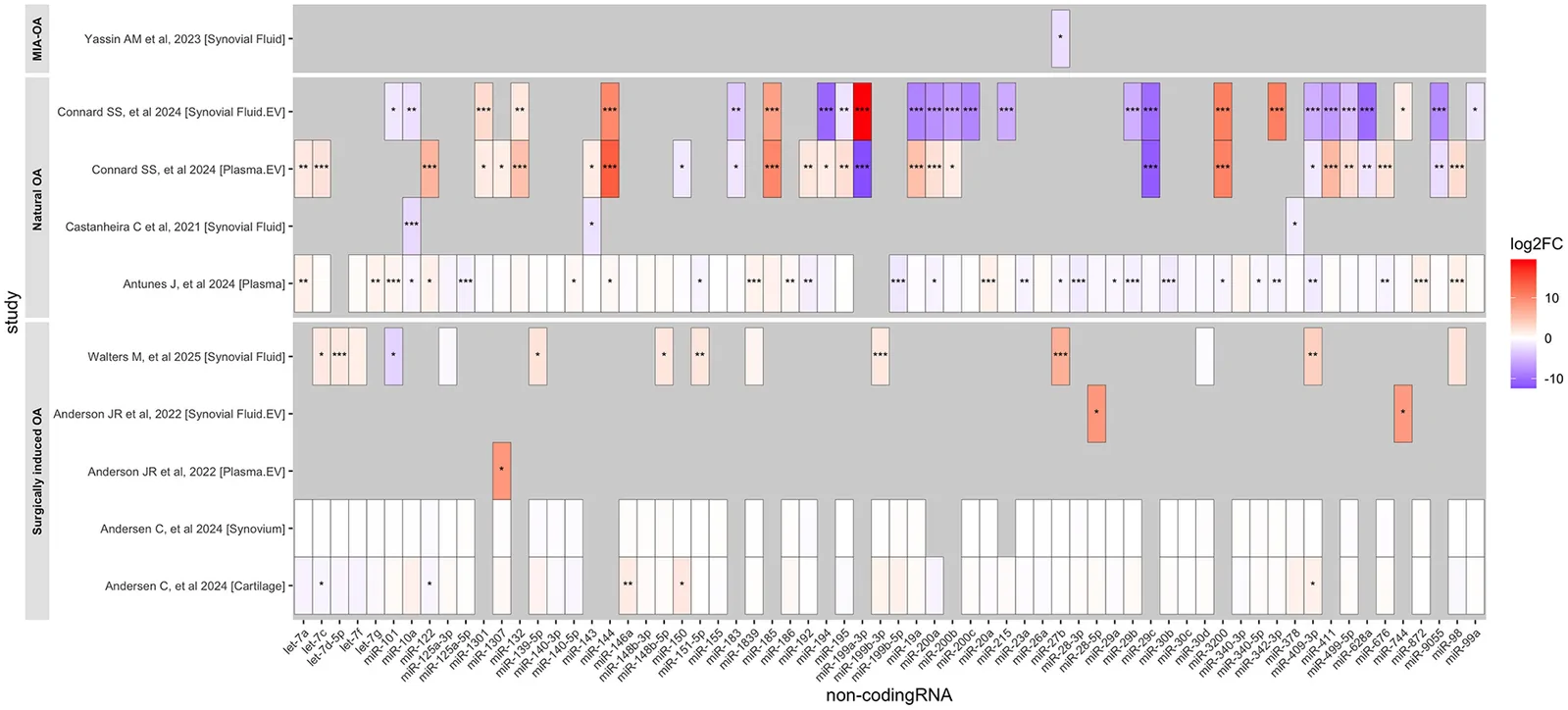
Heatmap of ncRNAs expressed in three or more samples across seven studies. Colors represent log_2_ (fold change) in expression (red, upregulated; blue, downregulated; white, no change). Only genes with detectable expression in ≥3 samples were included. Statistical significance of the differential expression between normal and OA joints is indicated by asterisks: *p* < 0.05 (*), *p* < 0.01 (**), and *p* < 0.001 (***). *P*-values are shown as reported in the original studies and are presented for descriptive purposes only.

**Figure 3:**
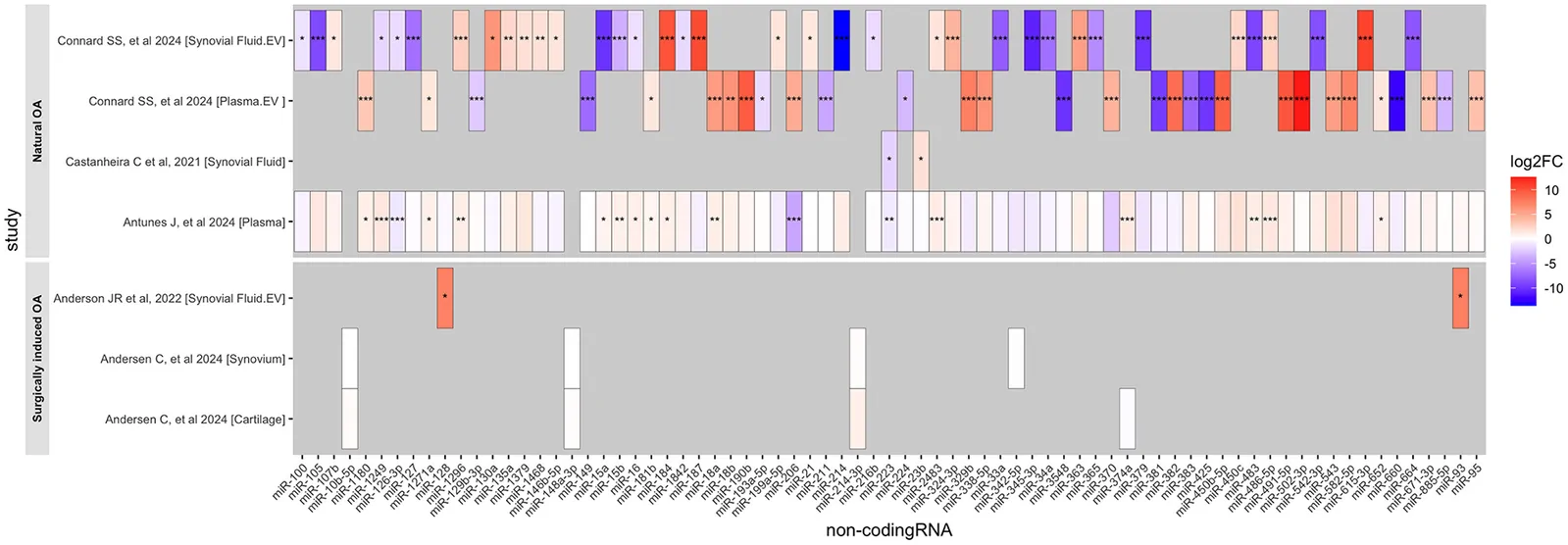
Heatmap of ncRNAs expressed in two samples across five studies. Colors represent log_2_ (fold change) in expression (red, upregulated; blue, downregulated; white, no change). Only genes with detectable expression in 2 samples were included. Statistical significance of the differential expression between normal and OA joints is indicated by asterisks: *p* < 0.05 (*), *p* < 0.01 (**), and *p* < 0.001 (***). *P*-values are shown as reported in the original studies and are presented for descriptive purposes only.

**Figure 4:**
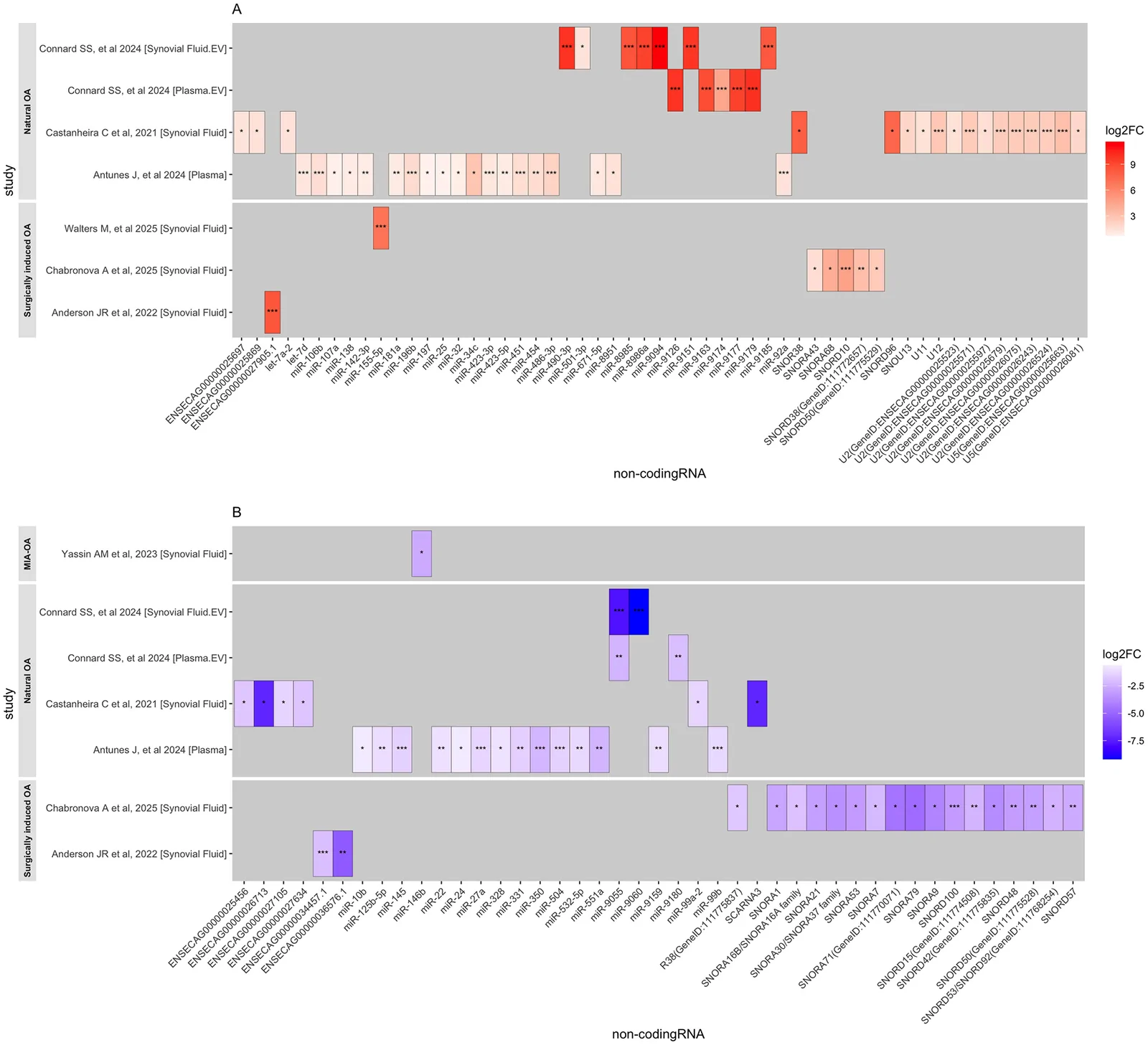
Heatmap of ncRNAs with detectable expression in one unique study that are significantly upregulated or downregulated. Colors represent (Log_2_fold change) in expression [red, upregulated **(A)**; blue, downregulated **(B)**]. Statistical significance of the differential expression between normal and OA joints is indicated by an asterisk: *p* < 0.05 (*), *p* < 0.01 (**), and *p* < 0.001 (***). *P*-values are shown as reported in the original studies and are presented for descriptive purposes only.

Analysis of the dataset revealed several classes of ncRNAs, including micro-RNAs (miRNAs), small nucleolar RNAs (snoRNAs), small nuclear RNAs (snRNAs), and long non-coding RNAs (lncRNAs). MiRNAs constituted the largest fraction of ncRNAs identified. Among the ncRNAs identified in three or more samples across studies, 237 were miRNAs, 102 of which were significantly differentially expressed in at least one sample type across these studies (Figure 2). Among ncRNAs identified in two samples across studies, 150 miRNAs were identified, 88 of which were significantly differentially expressed in at least one sample type across these studies (Figure 3). There were 59 miRNAs, 26 snoRNAs, 12 snRNAs and 2 lncRNAs with significant relative expression of ncRNAs in one sample type in a single study (Figures 4A, 4B).

### 3.7 Comparison of differential expression of ncRNA across studies

All differential expressions presented are in reference to control samples.

#### 3.7.1 Comparison of differential expression of ncRNAs expressed in three or more samples across studies

A heatmap was used to visualize the ncRNAs that were expressed in at least three sample types, with at least one sample significantly differentially expressed, across seven studies (Figure 2). Among these, let-7a, miR-1307, miR-144, miR-744, and miR-98 were upregulated in at least three sample types across two separate studies. Let-7a was significantly upregulated in natural OA in plasma (35) and plasma-derived extracellular vesicles (EVs) (37), however it was identified but not significantly up- or downregulated in surgically induced OA in synovium (15) and cartilage (15) (Figure 2). MiR-1307 was increased in plasma EVs in surgically induced OA (32) and natural OA (37), and was identified but not significantly increased in plasma (35), synovium (15), and cartilage (15) in OA samples (Figure 2). MiR-144 was significantly increased in two studies of natural OA in synovial fluid EVs (37), plasma EVs (37), and in plasma (35) (Figure 2). MiR-744 was significantly increased in natural OA in synovial fluid EVs (37) and in experimentally induced OA synovial fluid EVs (32), and was identified but not significantly increased in plasma OA samples (35) (Figure 2). MiR-98 was significantly increased in natural OA in plasma EVs (37) and in plasma (35), and was identified in surgically induced OA in synovial fluid (33), synovium (15), and cartilage (15) samples (Figure 2).

MiR-10a was downregulated in natural OA in synovial fluid EVs (37), synovial fluid (36), and plasma (35) (Figure 2). MiR-29b was downregulated in synovial fluid EVs (37) and plasma (35) in natural OA individuals (Figure 2). Both miR-10a and miR-29b were identified but not significantly decreased in surgically induced OA synovium (15) and cartilage (15) samples (Figure 2).

In one study of natural OA, miR-1301, miR-132, and miR-185 were significantly upregulated in synovial fluid EVs and plasma EVs OA samples (37). These miRNAs were identified but not significantly upregulated in plasma from another natural OA study (35) (Figure 2). In the same study (37), miR-183, miR-29c, miR-628a, and miR-9,055 were significantly downregulated in synovial fluid EVs and plasma EVs, and again these miRNAs were identified but not significantly up- or downregulated in plasma samples from natural OA samples (35) (Figure 2).

Several ncRNAs such as let-7c, miR-101, miR-122, miR-143, miR-150, miR-151-5p, miR-192, miR-27b, miR-3200, miR-342-3p, miR-409-3p, and miR-676 exhibited oppositional expression depending on sample type and study (Figure 2). Within the same study (37), miR-194, miR-195, miR-19a, miR-200a, miR-200b, miR-411, and miR-499-5p were upregulated in plasma EVs from natural OA samples but were downregulated in synovial fluid EVs from OA samples. MiR-199a-3p followed the opposite expression pattern, with downregulation in plasma EVs but upregulation in synovial fluid EVs (Figure 2).

#### 3.7.2 Comparison of differential expression of non-coding RNAs expressed in two samples across studies

For studies in which the same ncRNAs were expressed in two samples, miR-1180, miR-1271a, miR-181b, miR-18a, and miR-652 were upregulated in plasma (35) and plasma EVs (37) in natural OA samples (Figure 3). MiR-1296, miR-184, miR-2483, and miR-486-5p were consistently upregulated in plasma (35) and synovial fluid EVs (37) in natural OA samples (Figure 3). In samples from natural OA, there was downregulation of miR-126-3p in plasma (35) and synovial fluid EVs (37). MiR-223 was downregulated in plasma (35) and synovial fluid samples from (36) natural OA samples (Figure 3). A subset of miRNAs, including miR-1249, miR-15a, miR-15b, miR-16, and miR-483, exhibited opposite expression patterns between the two samples, with upregulation in plasma from individuals with natural OA (35) and downregulation in synovial fluid EVs (37) (Figure 3). MiR-206 was upregulated in plasma EVs (37) and downregulated in plasma (35) (Figure 3).

#### 3.7.3 Comparison of differential expression of non-coding RNAs which were unique to one sample in a single study

Numerous ncRNAs were only found to be significantly upregulated in a single sample type derived from OA individuals from a single study (Figure 4A) Among these ncRNAs, some miRNAs show notably high fold change (log_2_ fold change >6). MiR-155-5p (33) was significantly upregulated in synovial fluid from surgically induced OA. Additionally, miR-490-3p, miR-8985, miR-8986a, miR-9094, miR-9151, miR-9185 were significantly upregulated in synovial fluid EVs from natural OA samples (37). In plasma EVs from OA samples from the same study, miR-9126, miR-9163, miR-9177, and miR-9179 also had significantly increased expression with large fold changes (37) (Figure 4A).

ENSECAG00000027905.1 (32) is a lncRNA with high relative expression (log_2_fold change: 8.4) in synovial fluid samples from induced OA individuals. Among the snoRNAs, several genes showed notable expression (log_2_fold change>3) in synovial fluid from natural OA, including SNOR38 and SNORD96 (36), as well as SNORA68, SNORD10, SNORD38 (Gene ID: 111772657) (34) in induced OA synovial fluid samples in their individual respective studies (Figure 4A). Among the snRNAs, multiple gene copies encoding U12, U2 and U5 were highly expressed (log_2_fold change>1) in synovial fluid from natural OA (36) (Figure 4A).

Additionally, there were various ncRNAs that were found to be downregulated in a single OA sample type from a single study (Figure 4B). Among miRNAs, ENSECAG00000026713 (36), miR-9055, and miR-9060 (37) were highly downregulated (log_2_fold change < −5) in synovial fluid and synovial fluid EVs in natural OA individuals. ENSECAG00000036576.1 (a lncRNA) and ENSECAG00000034457.1 (a snRNA) were downregulated in OA synovial fluid (32) (Figure 4B). Additionally, several snoRNAs demonstrated remarkable downregulation in OA: in natural OA, SCARNA3 was downregulated (log_2_fold change: −7.28) in synovial fluid (36), while in induced OA, SNORA79 (log_2_fold change: −4.8) and SNORA71 (GeneID:111770071) (log_2_fold change: −4.5) were significantly down regulated in synovial fluid (34) (Figure 4B).

## 4 Discussion

Identifying biomarkers for equine OA diagnosis and intervention prior to the onset of structural and functional changes is needed (38). NcRNAs have emerged as candidates for both the diagnosis of OA and as therapeutic targets (20, 39, 40). Efforts are increasing to identify ncRNAs associated with equine OA. This study represents the first systematic synthesis of existing literature comparing ncRNA expression profiles between healthy and OA-affected equine joints.

### 4.1 Role of differentially expressed ncRNAs across three or more samples in OA pathogenesis based on the human and animal model literature

Several ncRNAs were identified within three or more samples across independent studies of equine OA. Shared expression patterns were found in let7a, miR-1307, miR-144, miR-744, miR-98 with significant upregulation in two OA sample types from independent studies. MiR-10a and miR-29b had shared downregulation expression profiles in OA samples from two independent studies.

Some of these findings were in agreements with the mouse and human literature, including let-7a, miR-1307, and miR-144. Inhibition of let-7a in LPS-treated ATDC5 cells, an immortalized mouse cartilage line, resulted in increased proliferation, decreased apoptosis, and attenuated inflammatory responses (41) suggesting a potential role for let-7a in inflammatory regulation relevant to OA. Our findings of upregulated let-7a in multiple equine OA models are consistent with the possible contribution of let-7a to inflammation and supports these previous studies (35, 37). Elevated expression of miR-1307-5p was found in cartilage tissue of human OA patients and in the cartilage of an induced OA mouse model (42). The results of our systematic review showed increased expression of miR-1307 in plasma EVs from equine patients with OA (32, 37), which aligns with this previous research and highlights miR-1307's potential role in OA pathogenesis. An *in-vitro* study including SW1353 chondrocytes (human chondrocyte cell line) (43) suggested miR-144-3p may be related to OA pathogenesis, as it inhibits the expression of ADAMTSL3 mRNA in chondrocytes, which produces a protein involved in organizing the extracellular matrix. This proposed pathogenic involvement of miR-144 in OA is in alignment with our findings of increased miR-144 expression in OA tissues from two equine studies (35, 37).

Other equine ncRNAs differential expression patterns were in contrast with what has been previously found in human and mouse model literature. Specifically, miR-744, miR-98, miR-10a, and miR-29b followed opposite trends. MiR-744-5p was downregulated in serum from humans with OA and in an LPS-induced human chondrocyte model compared to controls, suggesting a potential role in reducing chondrocyte inflammation by targeting THBS2 (44). This contrasts with our equine findings, in which miR-744 was significantly increased in synovial fluid EVs from equine OA joints (32, 37). This review found that miR-98 was upregulated in equine OA samples compared to controls in two studies (35, 37). However, a previous human study (45) found that miR-98-5p was downregulated in isolated cartilage samples from human OA patients. In a study comparing chondrocytes from 10 OA human patients and 10 healthy controls (46), they found that miR-10a-5p is upregulated in OA. The study also utilized a mouse model and found miR-10a-5p expression was significantly increased in cartilage from anterior cruciate ligament transection (ACLT)-induced OA mice compared with sham-operated controls. Furthermore, miR-10a-5p levels were elevated in both human and mouse chondrocytes following IL-1β treatment, suggesting that its upregulation is associated with OA-related inflammatory conditions. In contrast, miR-10a was downregulated in natural OA equine samples from synovial fluid EVs, synovial fluid, and plasma (35–37) as reported in this review. It was also identified but not significantly increased or decreased in experimentally induced equine OA samples, which indicates a possible association with OA pathology. Previous studies indicated that miR-29b was upregulated in chondrocytes from human OA patients compared to non-OA controls, and this upregulation was associated with loss of the chondrocyte phenotype (47). This differs from the downregulation of miR-29b reported in equine OA samples in this review (35, 37).

### 4.2 Role of differentially expressed non-coding RNAs across two samples in OA pathogenesis

Findings from included studies showed that miR-1180, miR-1271a, miR-1296, miR-181b, miR-184, miR-18a, miR-2483, miR-486-5p, miR-652 were significantly upregulated, whereas miR-126-3p and miR-223 were significantly downregulated in OA joints compare to control joints across two samples in independent studies (35–37).

Several of the ncRNAs that were upregulated in association with equine OA followed the same trends as human and rat models, such as miR-1271, miR-181b, miR-18a, miR-486-5p, and miR-652-3p. Increased expression of miR-1271 was observed alongside an increase in apoptotic cells in human OA cartilage (48). MiR-181b was found to be upregulated in human OA chondrocytes (49). Expression of miR-18a in rat chondrocytes was induced in response to IL-1β through activation of NF-κB and accelerate OA progression *in-vitro* (50). Significant increases in miR-486-5p in human OA have been observed, which may inhibit chondrocyte proliferation through suppression of SAMD2 (51). MiR-652-3p showed higher expression in plasma from human with hand OA compared to controls (52). Consistent upregulation of these ncRNAs across multiple species indicates that further investigation into their role in OA is warranted.

Expression of some of the ncRNAs identified in two equine samples differed from human and mouse literature. MiR-1180-3p was found to be downregulated in blood samples from late-stage OA patients compared to early-stage OA in human patients (53), suggesting a negative association with severity of OA disease. Our review summary found upregulation of miR-1180 in the systemic circulation of natural OA equine patients compared to normal controls, however there was no comparison between OA patients to assess miR-1180 expression at different stages of severity. Another study (54) found upregulation of circulating miR-126-3p in two cohorts of OA human patients compare to controls in plasma samples, as well as upregulation in plasma samples in a mouse model with partial medial meniscectomy (PMX). By contrast, miR-126-3p was downregulated in this review (35, 37). Overall, these ncRNAs may contribute to OA pathogenesis, however, since their expression was observed in only two samples, further investigation is necessary to validate these results.

### 4.3 Non-coding RNA with opposite direction across multiple samples

Based on our review, several ncRNAs such as let-7c, miR-101, miR-122, etc. exhibited opposing patterns of differential expression depending on the reviewed study. These studies used a variety of tissues, and evidence indicates that ncRNAs exhibit cell-specific and tissue-specific expression patterns in OA (55, 56). Differences in disease stage and inherent biological heterogeneity among individual horses could also have contributed to these inconsistent expression profiles. In addition, experimental methods such as sample collection, and RNA extraction, purification, storage, handling and testing conditions can impact ncRNAs expression (57–59).

### 4.4 Non-coding RNA classes investigated

In this review, miRNAs were the most common ncRNA class identified. The predominance of miRNAs across multiple studies may be attributed to their involvement in modulating extracellular matrix deposition, chondrocyte apoptosis (60, 61), regulation of chondrogenic process (62), and effecting cartilage degradation and homeostasis (63). In addition, it may reflect their stability and detectability in all biological fluids (64).

Based on our review, beyond miRNAs, snoRNAs were the next most abundant ncRNAs differentially expressed in OA pathobiology. Several studies have reported differential expression of snoRNAs in mouse OA (65) and human OA (66, 67). Notably, recent evidence has highlighted a mechanistic role for snoRNA-mediated ribosome heterogeneity in driving OA development and progression in humans (26, 27). Furthermore, the involvement of snoRNAs in regulating ribosome heterogeneity has been described (34), representing the first study mapping temporal abundance patterns of snoRNAs in synovial fluid during OA development. It found temporal- specific expression patterns, with upregulation in early OA and downregulation in later stages, indicating potential differential roles over disease progression.

### 4.5 Osteoarthritis models

The use of experimentally induced OA models, such as the equine carpal osteochondral fragment model, is particularly valuable for ncRNAs studies. This model offers a controlled and reproducible system with a known time of OA onset and a single etiological factor, making it easier to observe ncRNAs expression changes related to disease progression and ultimately evaluate response to treatment (32–34). Since the aim of this study was synthesizing the ncRNAs expression profile differences between equine individuals with OA disease and healthy joints, rather than focusing on OA progression, the analysis utilized the last time point as a case and the first time point as a control for longitudinal studies. Accordingly, ncRNAs expressed only during middle time points were not included in analysis, which could be the reason for the higher number of ncRNAs observed in natural OA. The natural OA samples likely had some biologic variation in disease state and timeline compared with the surgically induced OA, resulting in a more diverse ncRNA profile.

Among ncRNAs identified in 3 or more samples, miR-1307 and miR-744 had shared expression profiles, with significant upregulation in both surgically induced OA and natural OA. Let7-a and miR-98 were only significantly upregulated in natural OA samples, however both were identified in samples from surgically induced OA. MiR-10a and miR-29b were only significantly downregulated in samples derived from natural OA and were detected but not significantly upregulated or downregulated in samples derived from surgically induced OA. MiR-144 expression was only found in samples of natural OA. While it is commons for our experimental models to fail to completely replicate the naturally occuring disease state, it is encouraging that some ncRNAs follow the same trends in both natural and surgically induced OA for disease marker identification.

The differential expression of specific ncRNAs identified in this review may reflect different underlying OA-related pathological processes in equine joint tissues compared to other experimental and naturally occurring disease models in other species. Although natural OA in horses is generally considered a good model for human OA (11), appropriate cross-species comparison depends on the type of OA being studied (degenerative joint disease leading to OA, post-traumatic OA, or rheumatoid OA) (68).

### 4.6 Non-coding RNAs expression platform

The methods for assessing ncRNAs expression in included studies were qPCR, and NGS. Our review highlighted that Castanheira et al. (36) and Walters et al. (33) performed RT-qPCR validation. Additionally, Antunes et al. (35) employed ddPCR validation following NGS. NGS provides non-biased comprehensive profiling of ncRNAs and is considered the gold standard for identifying tissue-specific ncRNAs, their targets and their interactions. Specific NGS methodologies, particularly library preparation steps such as ligation, reverse transcription, and amplification, can introduce biases which result in misrepresentations of true miRNA expression levels (69). Consequently, given the large number of candidates ncRNAs identified through sequencing further investigation of their biological relevance through targeted validation studies is recommended.

In our systematic review, there were discrepancies observed between ncRNA expression obtained by NGS and those validated with PCR (Table 4). Previous studies comparing the accuracy and sensitivity of different methods have reported inconsistent results. For example, in a study investigating plasma miRNA profiles in early-stage osteoarthritis and rheumatoid arthritis, RT-qPCR analysis showed that only six of the twelve miRNAs exhibited expression patterns consistent with the miRNA NGS results (70). Whereas in a study comparing the expression profiles of lncRNAs in the cartilage of seven patients with knee OA and six healthy individuals, six lncRNAs were randomly selected for RT-qPCR validation, and the results were consistent with the sequencing data (71). While qPCR assays have high sensitivity and specificity, their performance depends on the abundance of the target and the efficiency of amplification (72). If closely related sequences exist, there is an increased risk of false amplification (73, 74). In comparison with qPCR, NGS enables comprehensive analysis of entire target regions, allowing detection of known and novel genetic variants and simultaneous integration of multiple genomic regions. However, NGS approaches are generally more time consuming, costly, and require substantial analysis (75). Therefore, the choice between qPCR and NGS depends on the specific experimental objectives, including the need for targeted quantification vs. comprehensive variant discovery.

Beyond the studies included in the present systematic review, a study (76) investigated circulating and synovial fluid miRNAs in equine carpal OA. Although, this study was published outside the predefined search period and was therefore not included in our systematic review, its findings provide additional evidence regarding ncRNAs associated with equine OA. Using miRNAs sequencing, they found increased expression of miR-146a, miR-148a, miR-140-3p, miR-199a-3p, miR-27b, and miR-223 in serum. While miR-490-3p, miR-129b-3p, miR-8951, miR-483, miR-23b, miR-423-3p, let-7f, miR-98, miR-23a, were decreased in OA serum samples compared to control. In synovial fluid miR-99a, miR-10b, miR-151-5p, miR-342-5p, miR-211 were upregulated, but miR-23b, miR-27b, miR-206 and miR-423-5p were found to be downregulated in OA compared to control (*p* < 0.05).

In addition, subsequent qPCR validation of six selected miRNAs (miR-199a-3p, miR-148a, miR-99b, miR-146a, miR-423-5p, miR-23b) showed miR-146a displayed a trend opposite to that observed by sequencing in serum and miR-148a, miR-99b, miR-146a, miR-423-5p, and miR-23b showed expression trends that differed from the sequencing results in synovial fluid. However, the validation results were not statistically significant.

### 4.7 Study limitations

This review is limited by the small number of eligible studies (*n* = 8) and substantial heterogeneity across study designs, tissue types, OA models, platforms used, and joint types. All ncRNAs were reported in only one to five studies, limiting confidence in the reproducibility and generalizability of individual findings. Although a meta-analysis could formally assess differential ncRNAs expression between OA and normal joints while accounting for covariates, the small number of studies and inconsistent methods precluded a complete meta-analysis. Consequently, findings should be interpreted as hypothesis-generating rather than confirmatory.

### 4.8 Future research

This review serves to highlight similarities across ncRNA expression profiles in equine OA and informs potential targets for future study of miRNAs as disease markers and treatment targets. Additionally, expression profiles of other ncRNAs, such as snoRNAs, could yield further novel targets. NGS is recommended to promote further exploration of this area with qPCR confirmation, when possible, for the most robust data quality. Future studies should strive to report all detected ncRNA expression characteristics such as fold change, standard error, and *p*-value, as well as ncRNA isoforms.

However, there has been research progress on the role of non-coding RNAs in osteoarthritis (OA) in veterinary species, their application as regenerative therapies have not yet been established in veterinary clinical practice. Continued research is expected for the use of biofluid or tissue derived non-coding RNAs as biomarkers for the early diagnosis and prognosis of OA, as well as targeted therapeutic agents for the treatment of OA as its early stages.

## 5 Conclusions

There are no available sensitive and specific biomarkers for detection of the initial stages of equine OA for use in clinical practice. The ideal early biomarker of OA would be obtained through minimally invasive techniques and remain stable after sampling, and thus OA-associated expression profiles of ncRNAs present in synovial fluid and plasma are attractive biomarker candidates and treatment targets. The similar expression profiles of let-7a, miR-1307, miR-144, miR-744, miR-98, miR-10a, and miR-29b across multiple studies of equine OA highlight their potential as candidate diagnostic biomarkers and therapeutic targets in equine OA that require independent validation. To further our understanding of ncRNAs associated with equine OA, specific investigation should continue into these ncRNA candidates as well as continued exploration through less biased approaches such as NGS.

## Data Availability

The original contributions presented in the study are included in the article/supplementary material, further inquiries can be directed to the corresponding author/s.
